# *Aronia melanocarpa* Fruits as a Rich Dietary Source of Chlorogenic Acids and Anthocyanins: ^1^H-NMR, HPLC-DAD, and Chemometric Studies

**DOI:** 10.3390/molecules25143234

**Published:** 2020-07-15

**Authors:** Agnieszka Zielińska, Paweł Siudem, Katarzyna Paradowska, Małgorzata Gralec, Sławomir Kaźmierski, Iwona Wawer

**Affiliations:** 1 Department of Physical Chemistry, Physical Pharmacy and Bioanalysis, Faculty of Pharmacy, Medical University of Warsaw, Banacha 1, 02-097 Warsaw, Poland; agnieszka.zielinska@wum.edu.pl (A.Z.); katarzyna.paradowska@wum.edu.pl (K.P.); malgorzata.gralec@wum.edu.pl (M.G.); 2 Centre of Molecular and Macromolecular Studies, Polish Academy of Sciences, Sienkiewicza 112, 90-363 Łódź, Poland; kaslawek@cbmm.lodz.pl; 3 Herbology Department, Carpathian State University, Rynek 1, 38-400 Krosno, Poland; iwona.wawer@kpu.krosno.pl

**Keywords:** chlorogenic acid, anthocyanins, HPLC-DAD, ^1^H-NMR, *Aronia melanocarpa* fruits

## Abstract

*Aronia melanocarpa* (Michx.) Elliott’s (chokeberry) besides anthocyanins contains significant amounts of hydroxycinnamic acids: Chlorogenic and its isomer neochlorogenic acid. They exhibit antioxidant, anti-inflammatory, antidiabetic, and antibacterial activities, thus they can have a significant impact on the health-promoting properties of *Aronia*. The aim of our research was to determine the changes in the content of chlorogenic acids (CGAs) and anthocyanins during fruit development and ripening, with a particular emphasis on acids. *Aronia* fruit samples were collected from July to October on two organic farms in Poland. The chemical composition of the extracts was determined by NMR spectroscopy and HPLC-DAD. ^1^H-NMR and HPLC data were analyzed using chemometric analysis and multivariate statistics (PCA). The results showed that the content of chlorogenic acids and anthocyanins changes during ripening and depends on the time of harvest and the region of cultivation. A correlation between the time of CGAs reduction and the appearance of anthocyanins was also noticed. The result of our research was also a database in the form of NMR parameters, which allows analysis of the metabolite profile and tracking of its changes. The ^1^H-NMR spectrum showing anthocyanin and CGA resonance can be considered the *Aronia* berry fingerprint.

## 1. Introduction

Chlorogenic acids (CGAs) are phenolic acids that are derived from esterification of cinnamic acids with quinic acid. A number of structures, such as caffeoylquinic acids and dicaffeoylquinic acids, exist in several isomeric forms in plants [1]. These compounds protect plant tissues from damage by oxidative stress, pathogen infection, and wounds. They are also used to mediate animal and human health. CGAs exhibit multiple biological properties, including antioxidant, anti-inflammatory, antidiabetic, anticarcinogenic, and antibacterial activities. The effects of CGAs on different aspects of human health have been summarized and discussed [2,3]. The antiviral activity of chlorogenic acid against influenza A virus [4] and the possible application of CGAs as a natural anti-inflammatory agent for the treatment of acute pneumonia [5] are also worth mentioning.

The most popular source of CGAs in the diet and up to 90% consumption is from coffee, although CGAs are also present in beverages prepared from herbs, fruits (e.g., apples, berries), and vegetables. The most abundant CGA in green coffee beans is 5-CGA, which accounts for 76–84% of the total CGAs or ca. 10 g/100 g coffee beans. However, the reactions occurring during coffee roasting lead to thermal degradation of CGAs, so that up to 99% are lost in high roasted beans. Controversial results regarding the association between coffee drinking and the risk of cardiovascular disease as well as the influence of coffee on the endothelial function can be explained by different contents of chlorogenic acids [6]. Additionally, roasting coffee results in the generation of hydroxyhydroquinone. This compound increased the production of reactive oxygen species and inhibited the chlorogenic acid-induced restoration of the endothelial function [7].

Fruits and vegetables (potatoes, tomatoes, vegetable *Chicorium endivia*) are also among the main sources of dietary CGAs [1]. Apples are a rich source of CGA, with the core part having the highest level (2.10 mg/g of dry fruit) and the apple flesh (0.48 mg/g of dry fruit) [8]. The studies performed with a tea (*Artemisia annua*) containing CGAs showed a strong anti-inflammatory effect by decreasing the secretion of proinflammatory cytokines IL-8 and IL-6 in the cells. Neochlorogenic acid (nCGA) and its isomers were isolated from mulberry leaf (*Morus alba* L.) [9,10].

Considerable interest has recently been observed in the bioactivity of CGAs related to oxidative stress, since consuming CGAs and polyphenol-containing beverages can alleviate oxidative stress in various disease models.

*Aronia melanocarpa* (Michx.) Elliott’s (black chokeberry) is a member of the Rosaceae family, native to northeastern America. Nowadays, this plant is extensively grown in Europe, and cv. Nero is the most popular in Poland. *Aronia*, known as black chokeberry, contains a variety of valuable polyphenolic compounds with strong antioxidant properties. These include anthocyanins, catechins, procyanidins, and phenolic acids [11]. In comparison with other berries, *Aronia* contains significant amounts of hydroxycinnamic acids: Chlorogenic (35.5 mg/100 g) and its isomer neochlorogenic acid (21.5 mg/100 g), as established by Oszmiański and Sapis in 1989 [12]. The same authors also first reported on anthocyanins of *Aronia* [13]. In chokeberry fruits, the anthocyanins represent about 25% of the total polyphenols. Anthocyanins in *Aronia melanocarpa* are a mixture of four cyanidin glycosides: 3-galactoside, 3-glucoside, 3-arabinoside, and 3-xyloside, of which cyanidin 3-galactoside is the dominating one. Chlorogenic and neochlorogenic acids represent 7.5% of *Aronia* fruit polyphenols. Later results [14] confirmed the earlier reports.

The importance of antioxidant constituents of berries in the maintenance of human health and protection from coronary heart disease and cancer is raising the interest of food manufacturers and consumers. Because of their astringent taste, *Aronia* fruits are not eaten raw but are commonly used to produce juice or nectar, winning more consumer approval. The chemical composition of berries or freshly pressed juice distinguishes them from other berries by high contents of sorbitol and polyphenols. The cultivar, harvest time, habitat, maturation, and others affect their content. The total phenolic content of *Aronia* berries (in mg/100 g DW) was determined to range from 3440, 3760 (cultivar ‘Nero’), 4010, and 4210 (cultivar ‘Viking’), to as high as 7465 and 7849 [11].

*Aronia* fruits are known for their health benefits, and the juice and fruit extracts are recommended, among others, as dietary support for people with cardiovascular disease [15] and diabetes [16,17]. *Aronia* berries can be used in the diet as juice, or as dry extract in capsules, an ingredient of dietary supplements. *Aronia* extracts may help to reduce total serum cholesterol, LDL (low-density lipoprotein) cholesterol, and triglycerides in patients with metabolic syndrome as well as glucose levels in the blood [18,19,20].

The aim of our research was to determine the content of chlorogenic acids and anthocyanins during fruit development and ripening using HPLC (high-pressure liquid chromatography) and ^1^H-NMR (nuclear magnetic resonance) methods. NMR spectroscopy (techniques 1-D and 2-D) was used to identify individual components and to determine the profile of bioactive compounds in the fruit extract. Computer analysis of the position and intensity of the characteristic signals in the NMR spectra yields a multidimensional space, reflecting plant metabolism (metabolomics). In line with previous studies using principal component analysis (PCA) profiling [21], this approach provides a simpler analytical protocol to identify the geographical origin of a plant raw material. 

## 2. Results and Discussion

An important aim of this contribution was to collect ^1^H-NMR data for *Aronia* berries and to assess the value of PCA analysis. Detailed questions that require addressing are whether it is possible to distinguish between green and purple fruits according to several parameters, such as the time of collection, geographical origin, or growth conditions (temperature, altitude). For each PCA score plot, a PCA analysis produces a loading plot, in which data points that are responsible for the distinction are displayed. PCA analysis should provide unique molecular markers. The nature of these points in the loading plot and their utility as molecular markers unique to a sample were examined.

A series of methanolic extracts from 22 different berry samples (from green to dark purple) were analyzed. An optimized standard extraction method was used for the extraction process. The analytical methods (^1^H-NMR, HPLC) used allowed us to determine the presence of chlorogenic acids, i.e., CGA and nCGA, as well as four anthocyanin glycosides. The use of PCA allowed us to observe the differences and dependencies in the profile of the main components of chokeberry during ripening.

### 2.1. NMR Analysis of Chlorogenic Acids and Anthocyanins

The changes of line intensities in the ^1^H-NMR spectra allowed us to distinguish four main stages of fruit ripening. Therefore, one representative sample was selected for each stage, for which a full qualitative and quantitative analysis was carried out. ^1^H-NMR spectra were registered for the extracts of the fruits collected:

1. 29.05.2016 (green fruit; samples LS1–LS5);
2. 07.08.2016 (fruit turning pink, not yet fully ripe; samples LS6–LS8);
3. 18.09.2016 (ripe purple to black fruit, at harvest time; samples LS9–LS10); and
4. 30.10.2016 (overripe black fruits after the day of harvest; LS11).

**Figure 1:**
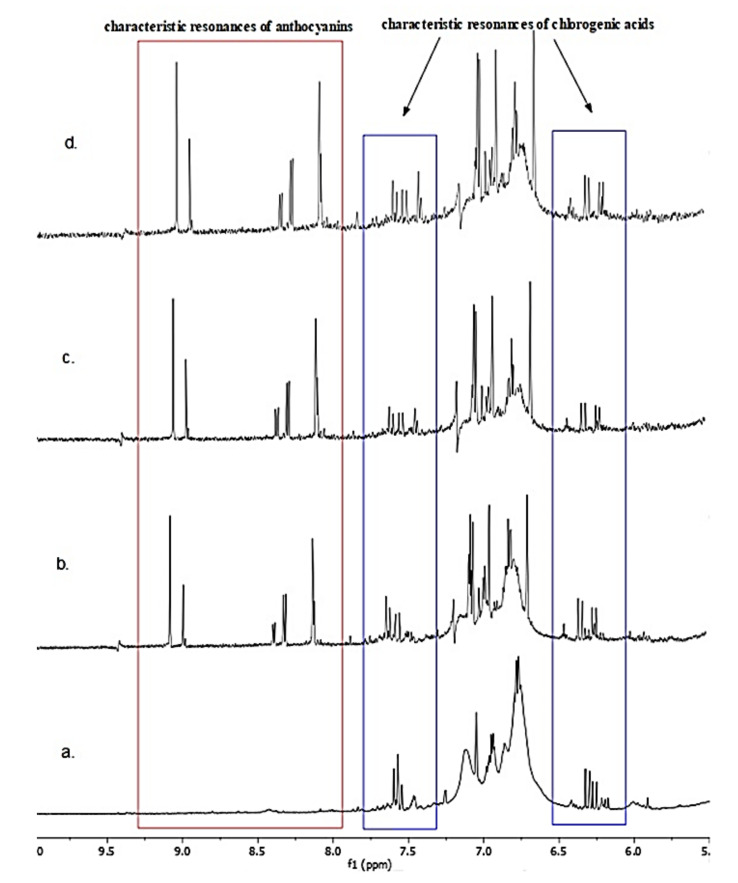
^1^H-NMR spectra of fruit samples from farm 1-LS (aromatic area from 5.5 to 10.0 ppm with diagnostic signals for anthocyanins (red frame) and chlorogenic acid (blue frame)): (**a**) LS1, (**b**) LS6, (**c**) LS9, (**d**) LS11.

^1^H-NMR spectra recorded for those berry extracts showed the resonances of sorbitol and other sugars, but remarkable differences appeared in the aromatic range, i.e., between 6 and 10 ppm (Figure 1).

In this range, there are characteristic signals of CGA and nCGA acids. The presence of both acids in ripe fruits and fruit juice was confirmed by Oszmianski and Wojdyło [14].

**Figure 2:**
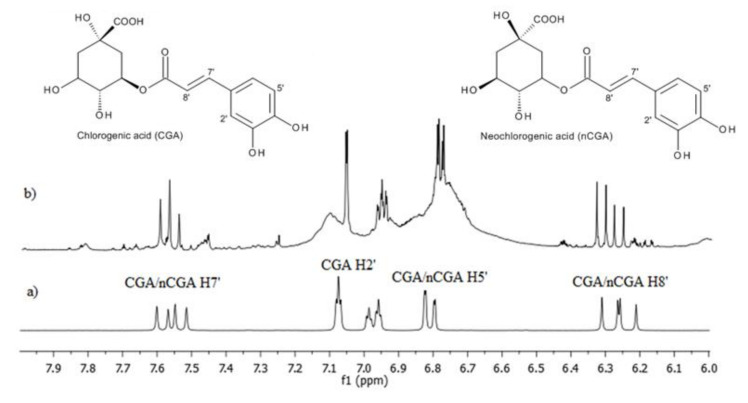
^1^H-NMR spectra of the mix chlorogenic acid and neochlorogenic acid (**a**) and for green fruit extract (**b**) with diagnostic signals, H5′, H2′, H7′, and H8′, respectively.

**Figure 3:**
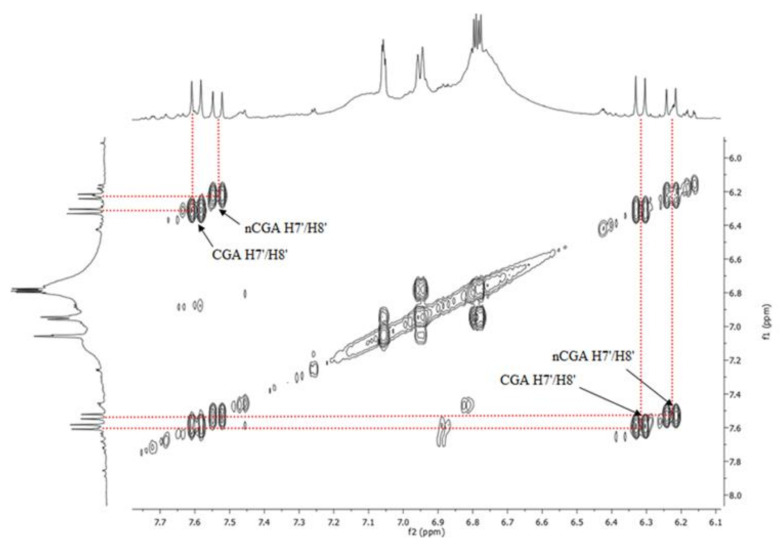
^1^H-^1^H COSY NMR spectrum for the green fruit extract showing H7′/H8′ correlations for CGA and nCGA.

Our research shows the presence of these acids in the fruit at every stage of ripening, as revealed by ^1^H-NMR spectra. The analysis of 1-D and 2-D ^1^H-NMR spectra for extracts made from green fruit showed a lack of anthocyanins at this stage of fruit development, but the resonances of chlorogenic acids are clearly visible. The ^1^H-NMR spectrum of green fruit extracts shows signals in the form of two doublets of different intensities in the aromatic region at δ 7.55 and 7.65 ppm (H7′) and at δ 6.20 and 6.35 ppm (H8′) (Figure 2b). In the 2-D spectra (^1^H-^1^H COSY) recorded for the same extract (Figure 3), there are correlations between H7′/H8′ for CGA and nCGA (slightly less intense), enabling fast and easy identification of both acids and quantitative analysis.

The signals arising from chlorogenic acid have higher chemical shift values than those from neochlorogenic acid, which was found previously by S.K. Wong et al. [22].

The intensity of these signals slightly decreases in the ^1^H-NMR spectra for the fruit collected at the beginning of August and is significantly lower for the fruit collected in September. The increase in their intensity is observed again in the case of fully ripe fruit (30.10.2016). However, this intensity is lower than for the green fruit extract (Figure 1). The chemical shift values (δ, ppm) for the diagnostic proton signals of the two CGAs are summarized in Table 1.

**Figure 4:**
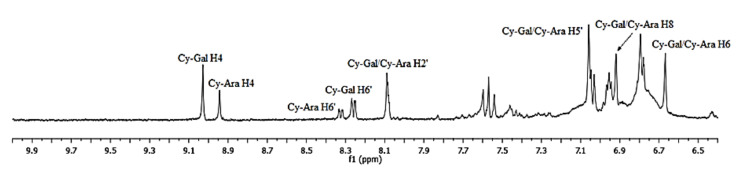
^1^H-NMR spectrum of sample LS6 (7th August) with diagnostic signals: H4, H6, H8, H2′, H5′, and H6’ for anthocyanins, cyanidin galactoside (Cy-Gal) and cyanidin arabinoside (Cy-Ara).

**Figure 5:**
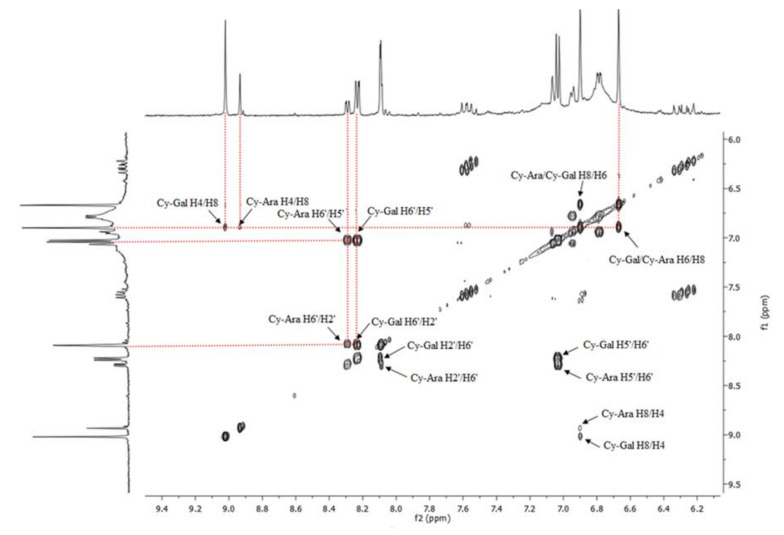
Two-dimensional (^1^H-^1^H COSY) NMR spectrum for fruit sample LS6 (7-August).

The ^1^H-NMR spectrum illustrated in Figure 4 shows distinct signals in the aromatic protons range at 6.5–9.0 ppm. Those signals did not appear in the spectra recorded for the green fruit extracts (first stage of growth). They are characteristic of anthocyanins, which start to appear in the second stage of fruit growth, together with a dark color. The 2-D ^1^H-^1^H COSY NMR spectrum for the ripe fruit extract showed H4/H8, H2′/H6′, and H5′/H6′ correlations (Figure 5) and the resonances of two main anthocyanins, cyanidin galactoside (Cy-Gal) and cyanidin arabinoside (Cy-Ara), can be easily recognized and assigned. Chemical shifts are collected in Table 2. The resonances of H4 and H6’ are well separated and can be used for quantification of these two anthocyanins.

### 2.2. HPLC Analysis of Chlorogenic Acids and Anthocyanins

**Figure 6:**
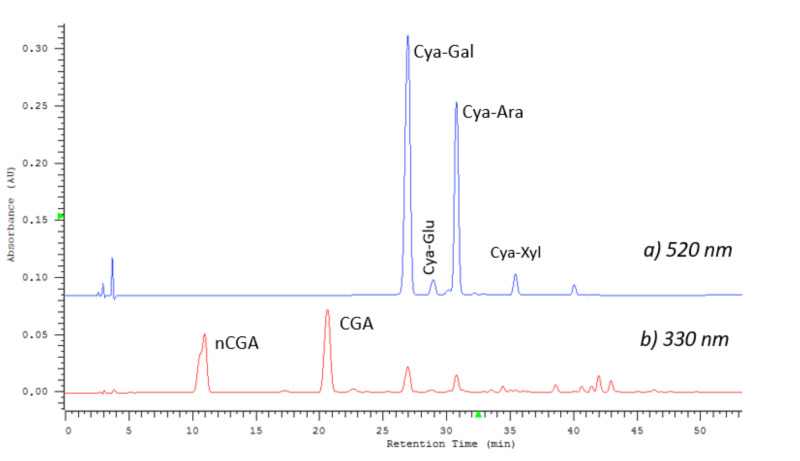
A typical HPLC chromatogram of *Aronia* anthocyanins (LE6 extract from August 7), registered at 520 nm (**a**). For comparison, the chromatogram at 330 nm (**b**) registered simultaneously for the same sample is shown below, with visible peaks of chlorogenic acids. Abbreviations: CyaGlu (cyanidin 3-glucoside), CyaXyl (cyanidin 3-arabinoside).

The chlorogenic acids and anthocyanin profiles of the examined extracts were characterized simultaneously by HPLC-DAD. Figure 6 shows the measurement result for a sample from August (LE6) containing both groups of compounds. A typical chromatogram of *Aronia* anthocyanins, recorded at 520 nm is presented in Figure 6a. Cyanidin 3-galactoside and cyanidin 3-arabinoside are the main anthocyanin dyes, with a small addition of glucoside and xyloside. Retention times were as follows: Cya-Gal, 26.8 min; Cya-Glu, 28.8 min; Cya-Ara, 30.7 min; and Cya-Xyl, 35.5 min. Simultaneously, the analysis allowed us to determine the content of chlorogenic acids, using the chromatogram registered at 330 nm (Figure 6b). The neochlorogenic and chlorogenic acid were detected at 10.9 and 20.5 min.

**Figure 7:**
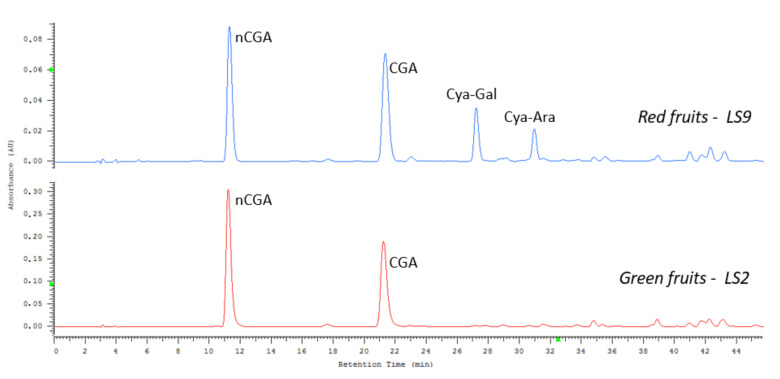
HPLC chromatograms of CGAs in typical green berries and red berries extracts at 330 nm; sample LS2, June and LS9, August.

The HPLC analysis allowed tracking of the changes in the profile of compounds during fruit ripening. Figure 7 shows a chromatogram registered at 330 nm, for the extracts from green and red fruits. The extract from June (LS2) contains only neo- and chlorogenic acids, whereas anthocyanins are visible in the chromatogram of red fruits (LS9, August).

**Figure 8:**
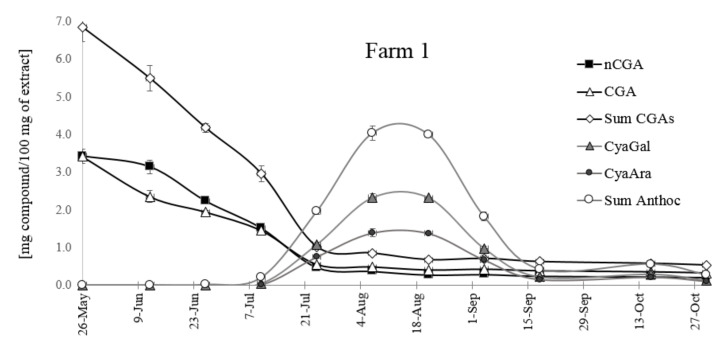
The changes of the CGA and anthocyanin contents from May to October in the fruit from central Poland (farm 1).

**Figure 9:**
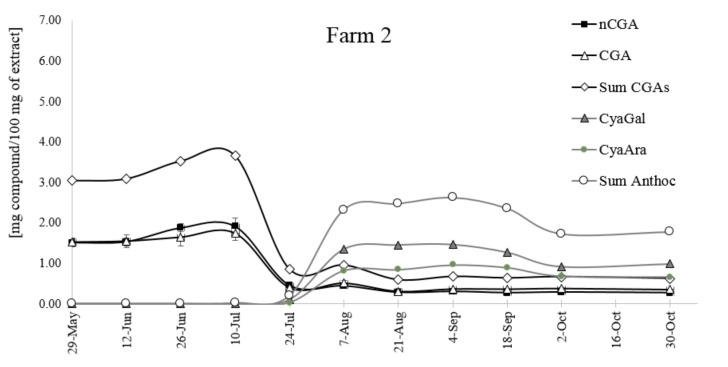
The changes of the CGA and anthocyanin contents from May to October in the fruit from the higher altitude location (farm 2).

The changes of CGAs and anthocyanins concentration in *Aronia melanocarpa* fruits were observed during seasonal growth and ripening (Figure 8 and Figure 9). In green fruits, the content of CGAs was very high; later, it decreased and remained unchanged to the end of the season (October). There was no significant difference in the content of two chlorogenic acids, although the amount of nCGA was higher than that of CGA, especially in green fruits. Two major isomers, 5-CQA and 3-CQA, were accumulated very early and within a short period, with the content of both isomers reaching 7 mg/100 mg of the extract (fruit from Farm 1) and only 3 mg /100 g in the fruit from the mountainous plantation (farm 2). Detailed data of the extracts’ composition are collected in Table S1.

The differences in the pattern of changes in the anthocyanin content for both farms are also visible. The level of anthocyanins in fruits growing in the mild climate of central Poland increased until mid-August, up to ca. 4 mg/100 g of the extract, and then it decreased. The optimum time for harvesting anthocyanin-rich fruits is between 4-August and 18-August. The fruits from farm 2 located at a higher altitude accumulate less anthocyanins (below 3 mg/100g of the extract), but the decrease of their content is slower. The harvesting period is longer, from 7-August to 18-September, and overripe fruits collected at the end of October still contain ca. 2 mg of anthocyanins per 100 g of the extract. It is interesting to note that the time of CGAs reduction correlates with the appearance of anthocyanins.

The above results suggest that climatic conditions (temperature, altitude) are a key environmental parameter that directly affect the CGAs’ and anthocyanins’ metabolism. The influence of the mean daily temperature on the sugar and CGA composition was demonstrated for coffee seeds [23]. Two major isomers, 5-CQA and di3,5-CQA, accumulated in coffee seeds, and this biosynthetic step was followed by a decrease in their relative content (mostly because the CGA synthesis reaches a plateau) and partial remobilization towards lignin biosynthesis. Their maximum transitory content was highly variable, and a delay in the biosynthesis peak was detected among locations. A dramatic impact of air temperature on the biosynthesis of different isomers during fruit growth was detected at different levels. Firstly, the delay in 5-CQA accumulation was temperature dependent, and it occurred in cool locations. The highly significant negative correlation between the 5-CQA content and temperature was demonstrated. This is one of the most important findings of the CGA profiling work: Warm climates trigger early accumulation of major CQA isomers, namely 5-CQA and di3,5-CQA, and favor their subsequent isomerization towards minor isomers during late stages. 

### 2.3. PCA Analysis of Chlorogenic Acids and Anthocyanins

**Figure 10:**
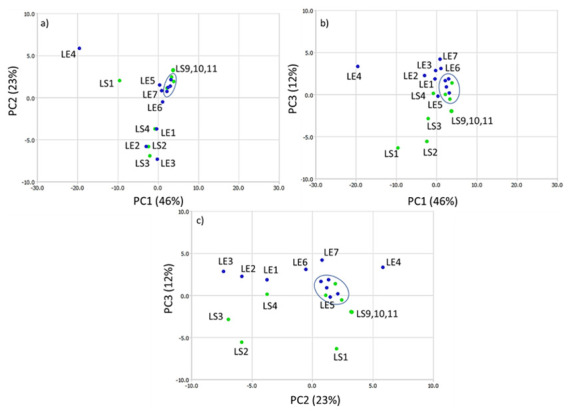
Score plots of principal component analysis of *Aronia* extracts: (**a**) PC1 versus PC2 score, (**b**) PC1-versus PC3 score, (**c**) PC2-versus PC3 score. Green points—the farm from central Poland, blue points—higher altitude location. On each plot, circled points correspond to ripe fruits.

PCA analysis was performed on the data obtained from NMR spectra after normalization. PCA plots are shown in Figure 10, where the first principal component (PC1) describes 46%, the second (PC2) 23%, and the third one 12% of the total variance. More than 80% of the total variance is explained by the first three PCs; therefore, only PC1, PC2, and PC3 were taken for further analysis.

Each PC plot shows that ripe fruits (circled on the plots) have lower internal group variance than the variance for unripe fruits. The points are separated mainly among PC1 (plot a and b) and PC3 (plot c). The samples from the farm in central Poland (green points) from 24-July (LS5) to 4-September (LS8) have lower group variance. They are clustered on the plots with samples LE8–LE11 (blue points, 4-Sepember–30-October). This group corresponds to ripe fruits in the harvest time and is circled on each plot. Based on PCA scores analysis, it seemed that the fruits from central Poland (farm 1) need less time to reach the composition of bioactive compounds typical of ripe fruits.

The HPLC analysis yielded similar results. The presence of anthocyanins is typical of ripe fruits and sample LS5 has a high anthocyanin concentration (Figure 10).

Our results suggest that the fruits from the plants cultivated at the higher altitude location need more time for ripening and the level of anthocyanins is lower than in the fruits from central Poland. However, the anthocyanins content from the higher altitude location does not decrease in ripe and overripe fruits. Therefore, LE8–LE11 are clustered together (also with LS5–LS8). PCA analysis of the variance shows that the group LS9–LS11 is different from the circled one. We observed a decrease of the anthocyanins level from 18th September and their low concentration in the fruits from central Poland.

**Figure 11:**
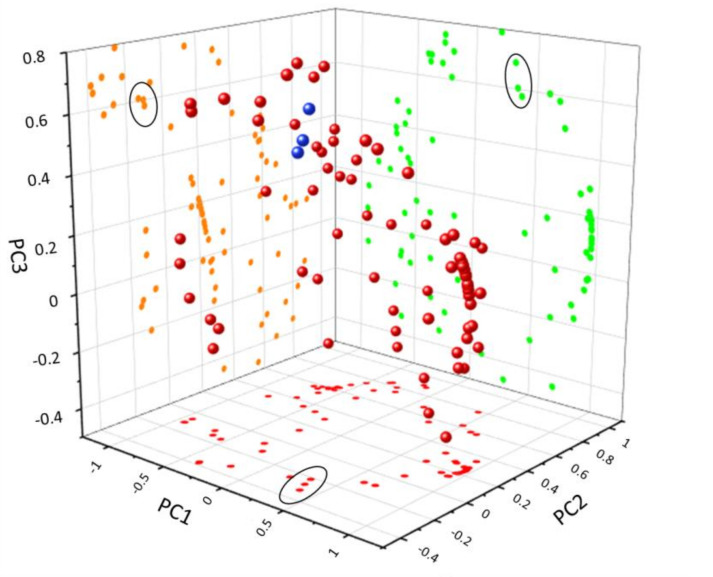
Loading plots based on PC1, PC2, and PC3 components; blue spheres and circled points refer to chemical shifts of anthocyanins.

Figure 11 shows that the samples from both farms are separated among PC3 (positive values of PC3 for the farm in the higher altitude location and negative for the farm in central Poland). Further details arise from the analysis of the PC’s loadings of the PC1–PC3 components (Figure 11).

The points on each loading plot were divided into three groups: I (δ 5.8–6.5 ppm), II (δ 6.5–7.6 ppm), and III (δ7.6–9.15 ppm). In the PC1-PC2 loading plot, the majority of the I group points are located in the fourth quarter and they do not affect the position of the sample within the PC plot [24]. However, some of them and most of the II group’s loadings are located in the third quarter and are associated with the position of samples LE1–LE3 and LS2–LS4. These loadings correspond to δ 6.2–7.6 ppm, the range characteristic of the CGA’s signals. On the PC1–PC3 plot, loadings characteristic of CGA’s signals are located in the first and third quarters, and on the PC3–PC2 plot, in the first, third, and partially in the second quarters. In each case, they are associated with the samples LE1–LE3 and LS2–LS4. The scores’ positions reflect a different content of the CGAs in the fruits collected before ripening, e.g., when the concentration of CGAs decreases.

In the last group, δ7.6–9.15 ppm, the position of the sample on the PC plot is determined by anthocyanins (δ 8.1 ppm Cy-Gal/Cy-Ara H2′, δ 8.25 ppm Cy-Gal H6′, δ 8.33 ppm Cy-Ara H6′, δ 8.95 Cy-Ara H4, and δ 9.05 Cy-Gal H40 (Figure 4). On each loading plot, they are marked by an ellipse. The loading plots of PC1, PC2, and PC3 confirm that the differentiation of the samples is possible based on the CGA and anthocyanin level and both types of compounds may be used as a ripening indicator. Four compounds were found to be the most important for the differentiation: Chlorogenic acid, neochlorogenic acid, and two anthocyanins, cyanidin galactoside and cyanidin arabinoside. These compounds are easily identified in the fingerprint ^1^H-NMR spectra. ^1^H-NMR is a fast and effective technique to create a fingerprint of *Aronia* berries, distinguishing them according to the producer regions.

## 3. Materials and Methods 

### 3.1. Plant Material 

Fruits of *Aronia* were collected during one season from May to October 2016 from two commercial plantations. Farm 1 is located in central Poland (average altitude of the plantation ca. 90 m above sea level), while farm 2 is situated in southern Poland (Klodzko Valley, average altitude of the plantation ca. 450 m above sea level), 400 km away from farm 1. The age and area of both plantations were similar (ca. 10 years and 5 ha).

Plant material was identified by Mr. Piotr Eggert (MA). The voucher specimens were deposited at the herbarium of the Department of Biology and Pharmaceutical Botany, Faculty of Pharmacy, Medical University of Gdańsk, Poland.

### 3.2. Collection, Storage, and Extraction of Fruit Samples 

The fruits were collected from the moment they were green and shaped until full maturity (at harvest time) and later, i.e., for a period of five months. Harvesting was performed every two weeks, collecting about 250 g of fruit from selected, healthy, and well-defined shrubs on the plantations. The berries were frozen, stored at −80 °C, and later freeze-dried. The extracts were prepared from powdered berries using acidified 80% MeOH. Eleven samples (Table 3) were available from one plantation and were subjected to analysis.

### 3.3. Determination of CGAs and Anthocyanins by HPLC-DAD

The samples of extracts (av. 20 mg) were dissolved in the following mixtures: Green fruits—4 mL of MeOH and 1 mL of 6% formic acid; and ripe fruits—4 mL of 6% formic acid and 1 mL of MeOH. Acidified solvent was used to prevent anthocyanins’ decomposition. The solutions were analyzed within a few hours of preparation. The contents of chlorogenic acids (CGA, nCGA) and cyanidin glycosides: 3-galactoside (CyaGal), 3-glucoside (CyaGlu), and 3-arabinoside (CyaAra), were analyzed by reversed-phase high-performance liquid chromatography with UV/VIS diode array detection (HPLC-DAD-UV/VIS). A Hitachi Chromaster HPLC system (Tokyo, Japan) was used, equipped with a gradient pump, a photodiode array detector, a column oven, and an autosampler with a thermostat. DAD detector specification: single beam photometric system (dispersion by diffraction grating); light source: D2 and W lamp, detecting element: 1024-bit photodiode array. The chromatographic separations were performed on a Merck Purospher STAR RP-18e column (5 μm, 250 mm × 4.6 mm) at 35 °C. The temperature of the autosampler was set at 10 °C.

The HPLC method enables simultaneous detection of chlorogenic acids and anthocyanins. A mobile-phase gradient system consisting of 4.5% (*v*/*v*) formic acid (A) and acetonitrile (B) was employed for the analysis. The gradient conditions were as follows: 0–5 min, 5% B; 5–15 min, 5–8% B; 15–50 min, 8–25% B; 50–55 min, 25–50% B; 55–65 min, 5% B, flow rate: 1–15 min, 1 mL/min; 15–50 min, 0.8 mL/min; and 50–65 min 1 mL/min. The chromatograms of anthocyanins were recorded at 520 nm, and the chromatograms of CGAs at 330 nm. The concentrations of the compounds were determined using an appropriate calibration curve (Table 4). The content of CyaXy (cyanidin 3-xyloside) was expressed as CyaAra. All measurements were performed in triplicate.

The linearity of the method was assessed by the value of the correlation coefficient (R^2^) of the calibration curve obtained for each standard (Table 2). A range of concentrations was prepared from stock solutions (1 mg/mL of every standard). The samples were subjected to HPLC analysis, in triplicate. Average peak area data were plotted against the corresponding standard concentrations to obtain the standard calibration curve.

The limit of detection (LOD) and limit of quantification (LOQ) concentrations were set using equations, where σ is the standard deviation of the y-intercept and S is the slope of the calibration curve: LOD = 3.3 × σ/S, LOQ = 10 × σ/S (Table 4).

The method precision was determined by injecting three concentrations of standards: 10, 100, and 200 µg/mL for Cya-Gal; and 50, 100, and 200 µg/mL for CGA, five times into the HPLC. Then, the relative standard deviation (RSD) values were calculated for all concentrations (Table S2, see Supplementary Material). For intraday and interday precision assessment, these concentrations were measured three times on the same day (at 0, 3, and 6 h) and on the next day. Anthocyanins are very sensitive to high temperatures and are easily degraded [25], so they should be stored in the fridge for a short time, and frozen for a longer time, preferably in dry form.

The method trueness was evaluated on recoveries, measured after spiking two samples of extracts (from green and red fruits), with a mixture of nCGA, CGA, Cya-Gal, Cya-Ara, and Cya-Glu standards (0.020 mg/mL of every compound). Recovery percentages (%R) were calculated according to the following equation:

%R = [(C_1_ − C_2_)/C_3_] × 100, (1)

where C_1_ (mg/mL) is the concentration of the fortified samples, C_2_ (mg/mL) is the concentration of the standard in samples before spiking, and C_3_ (mg/mL) is the concentration of the standard added to the sample. The results are presented in Table S3 (see Supplementary Material). Then, the samples were stored in a tightly capped volumetric flask at a temperature of 5 °C for 24 h and analyzed again to assess the solution stability.

The precision study results showed low values of % RSD (<2) for inter- and intraday variation, which suggested a good precision of the method. The recovery results were within the acceptable limit (recovery ranged from 98 to 103%) of the interday and intraday variation, which indicated that the method is accurate. There was no significant change observed for the chromatograms of the standard solution and the experimental solution. 

### 3.4. ^1^H-NMR Measurements 

^1^H-NMR spectra were recorded on a Bruker Avance III 600 spectrometer (Bruker BioSpin, Rheinstetten, Germany), operating at 600.13 MHz for ^1^H. The spectrometer was equipped with a 5-mm BBFO probe head. The temperature was set at 295 K during all measurements. The spectra were recorded using 5-mm NMR tubes (Norell) and methanol-d4 (Armar Chemical, Döttingen, Switzerland) as a solvent with the addition of 1M HCl (2.5 mL of 1M HCl for 50 mL methanol-d4). The residual signal of methanol (δ^1^H = 3.31 ppm) was used for chemical shift calibration. For each sample, a standard ^1^H spectrum and a spectrum with water signal presaturation were acquired. The frequency sweep was set at 12.33 kHz (20.5 ppm) and 256 scans of 64 K data points (TD) each were acquired with a relaxation delay (D1) of 1 s, acquisition time (AQ) of 2.65 s, and π/2 pulse duration of 11.00 µs. FIDs were processed with 0.3 Hz line broadening (LB = 0.3) without zero-filling (SI=64K). In order to obtain the standard ^1^H spectrum, the zg30 original pulse program was used and for the spectra with presaturation, the zgpr pulse program (Bruker library, Rheinstetten, Germany). The spectra were obtained and processed using TopSpin 3.1 program (Bruker BioSpin Rheinstetten, Germany) running under Windows 7 (64 bit) OS on the HP Z700 workstation.

### 3.5. Data Analysis

NMR-based metabolomics are frequently combined with chemometric analysis, like principal components analysis (PCA). PCA was performed by analyzing a set of ^1^H-NMR spectra. The chemical shift interval between δ5.80 and 9.15 ppm in the spectrum was divided (bin width 0.04 ppm) using MestReNova 11 software (Santiago de Compostela, Spain). All spectra were normalized. 

PCA was carried out using Statistica and Origin Pro 2019 Network software (Northampton, MA, USA). PCA was used to visualize and overview the data, reduce the number of variables, and to observe the separation between groups. The results of the analysis were displayed graphically as a scores plot. The plots were used to observe grouping in the data sets. All HPLC samples were analyzed in triplicate, and the results were expressed as the mean ± standard deviation (SD). Data were evaluated using Statistica 10 (StatSoft Inc., Tulsa, OK, USA) software. One-way analysis of variance (ANOVA) was applied with Tukey test for significance *p* < 0.05. The error bars represent the range of analytical replicates, *n* = 3.

## 4. Conclusions

The combined NMR and HPLC analysis of hydroxycinnamic acids present in the fruits of *Aronia melanocarpa* showed that their content changes during ripening and depends on the time of harvest and the region of cultivation. A correlation between the time of CGAs reduction and the appearance of anthocyanins was also observed. Our results indicated that the cultivation located at a low altitude above sea level, with a mild climate (in our case in central Poland) has more suitable conditions for chokeberry growing that produces fruit with a high content of chlorogenic acid and anthocyanins.

The result of our research is also a database in the form of NMR parameters, which allows one to analyze the metabolite profile and track its changes. This profile is a specific fingerprint of a given chokeberry extract. The chlorogenic acid profile and its variability during fruit development is associated with the anthocyanin content, regardless of the place of cultivation. The use of NMR spectroscopy in combination with chemometric methods (PCA) to determine the composition of the extracts is still a new and non-standard approach, and it is a very useful tool for qualitative analysis of plant metabolites. 

In summary, not only anthocyanins as a verifying factor but also chlorogenic acids can be used to standardize and assess the quality of chokeberry fruit.

## Figures and Tables

**Table 1:** ^1^H-NMR chemical shifts (δ, ppm) for chlorogenic acid and neochlorogenic acid in MeOD-d4.

Chlorogenic Acid	δ ppm	Neochlorogenic Acid	δ ppm
H2′	7.06	H2′	7.05 (m)
H5′	6.80; 678 (d)	H5′	6.79; 6.78 (d)
H6′	6.95; 6.96 (dd)	H6′	6.95; 6.96 (dd)
H7′	7.61; 7.58 (d)	H7′	7.55; 7.52 (d)
H8′	6.33; 6.30 (d)	H8′	6.24; 6.22 (d)

**Table 2:** ^1^H-NMR chemical shifts (δ, ppm) for cyanidin galactoside (Cy-Gal) and cyanidin arabinoside (Cy-Ara) in MeOD-d4.

	Cy-Gal, δ ppm	Cy-Ara, δ ppm
H2′	7.06	7.05 (m)
H5′	6.80; 6.78 (d)	6.79; 6.78 (d)
H6′	6.95; 6.96 (dd)	6.95; 6.96 (dd)
H7′	7.61; 7.58 (d)	7.55; 7.52 (d)
H8′	6.33; 6.30 (d)	6.24; 6.22 (d)

**Table 3:** Numbering of the *Aronia* berry samples collected for analysis from two Polish farms.

Date of Collection	May 26	Jun 12	Jun 26	Jul 10	Jul 24	Aug 7	Aug 21	Sept 4	Sept 18	Oct 16	Oct 30
Farm 1	LS1	LS2	LS3	LS4	LS5	LS6	LS7	LS8	LS9	LS10	LS11
Farm 2	LE1	LE2	LE3	LE4	LE5	LE6	LE7	LE8	LE9	LE10	LE11

**Table 4:** Calibration curves, concentration range, limit of detection (LOD), and limit of quantification (LOQ) for standards.

Compound	Calibration Curve ^a^	R^2^	Linear Range (mg/mL)	LOD (mg/mL)	LOQ (mg/mL)
nCGA	A=25925751c − 27059	0.999	0.025–0.300	0.0005	0.0018
CGA	A=26805759c − 33058	0.999	0.025–0.300	0.0006	0.0018
Cya-Ara	A=26576879c + 2711	0.999	0.020–0.250	0.0003	0.0009
Cya-Gal	A=32796479c − 9427	1.000	0.005–0.250	0.0002	0.0006
Cya-Glu	A=40070383c − 7322	0.999	0.005–0.250	0.0002	0.0006

^a^ A—peak area (mAU), c—concentration (mg/mL) of the compound.

## Supplementary Materials

The Supplementary Materials are available online.
