# Supplementary material for: Aronia melanocarpa Fruits as a Rich Dietary Source of Chlorogenic Acids and Anthocyanins: 1H-NMR, HPLC-DAD, and Chemometric Studies
Source: Molecules. 2020 Jul 15;25(14):3234. doi: 10.3390/molecules25143234 (PMC7397235; doi:10.3390/molecules25143234)
Supplement: Supplementary file 1 [file molecules-25-03234-s001.pdf]

Supplementary Materials:

Table 1. S. Contents of chlorogenic acids and anthocyanins in extracts [mg compound/100 mg of extract]; different letters mean statistical difference by Tukey's test ($p < 0.05$).

		nCGA	CGA	Sum of CGAs	CyaGal	CyaGlu	CyaAra	CyaXy	Sum Anthoc
LS 1	26-May	3.43 ± 0.19	3.42 ± 0.19	6.850	ND	ND	ND	ND	ND
LS 2	12-Jun	3.14 ± 0.18	2.35 ± 0.16	5.491	ND	ND	ND	ND	ND
LS 3	26-Jun	2.24 ± 0.07	1.94 ± 0.05	4.175	ND	ND	ND	ND	ND
LS 4	10-Jul	1.51 ± 0.13	1.45 ± 0.08	2.963	0.044^{a} ± 0.004	0.004 ± 0.000	0.016 ± 0.002	ND	0.063
LS 5	24-Jul	0.47^{a,b} ± 0.02	0.54^{a} ± 0.03	1.014	1.081^{b} ± 0.048	0.049^{a} ± 0.003	0.755 ± 0.029	0.078 ± 0.003	1.963
LS 6	7-Aug	0.37^{a,b} ± 0.02	0.48^{a,b} ± 0.02	0.848	2.323^{c} ± 0.112	0.107 ± 0.004	1.39^{a} ± 0.066	0.223^{a} ± 0.009	4.043
LS 7	21-Aug	0.27^{b} ± 0.01	0.40^{a,b} ± 0.01	0.671	2.320^{c} ± 0.027	0.088 ± 0.001	1.378^{a} ± 0.013	0.213^{a} ± 0.013	3.999
LS 8	4-Sep	0.28^{b} ± 0.04	0.42^{a,b} ± 0.05	0.704	0.978^{b} ± 0.063	0.052^{a} ± 0.004	0.675 ± 0.042	0.115 ± 0.006	1.819
LS 9	18-Sep	0.24^{b} ± 0.01	0.38^{b} ± 0.02	0.621	0.220^{d,e} ± 0.023	0.015^{b,c} ± 0.002	0.155^{b} ± 0.018	0.027^{b,c} ± 0.003	0.417
LS 10	16-Oct	0.22^{b} ± 0.01	0.35^{b} ± 0.02	0.573	0.288^{d} ± 0.015	0.018^{b} ± 0.001	0.215 ± 0.014	0.035^{b} ± 0.002	0.557
LS 11	30-Oct	0.21^{b} ± 0.01	0.32^{b} ± 0.01	0.528	0.124^{a,e} ± 0.008	0.012^{c} ± 0.001	0.108^{b} ± 0.007	0.021^{c} ± 0.002	0.265
LE 1	26-May	1.51^{a} ± 0.09	1.53^{a} ± 0.09	0.067	ND	ND	ND	ND	ND
LE 2	12-Jun	1.54^{a} ± 0.10	1.55^{a} ± 0.16	0.079	ND	ND	ND	ND	ND
LE 3	26-Jun	1.88^{a} ± 0.10	1.65^{a} ± 0.22	0.003	ND	ND	ND	ND	ND
LE 4	10-Jul	1.92^{a} ± 0.20	1.75^{a} ± 0.19	0.014	ND	ND	ND	ND	ND
LE 5	24-Jul	0.45^{b} ± 0.06	0.40^{b} ± 0.05	0.026	0.1 ± 0	0 ± 0	0.03 ± 0	0 ± 0	0.13
LE 6	7-Aug	0.44^{b} ± 0.06	0.51^{b} ± 0.07	0.011	1.35^{a} ± 0.14	0.05^{a,b} ± 0.02	0.83^{a} ± 0.08	0.10^{a,c} ± 0.04	2.33
LE 7	21-Aug	0.28^{b} ± 0.07	0.31^{b} ± 0.08	0.000	1.46^{a} ± 0.30	0.06^{a} ± 0.01	0.85^{a} ± 0.12	0.11^{a} ± 0.03	2.48
LE 8	4-Sep	0.30^{b} ± 0.01	0.37^{b} ± 0.02	0.000	1.47^{a} ± 0.01	0.07^{a} ± 0.01	0.97^{a} ± 0.01	0.13^{b} ± 0.01	2.63
LE 9	18-Sep	0.27^{b} ± 0.01	0.37^{b} ± 0.01	0.000	1.27^{a} ± 0.05	0.05^{a,b} ± 0.00	0.90^{a} ± 0.05	0.13^{b} ± 0.01	2.36
LE 10	16-Oct	0.29^{b} ± 0.02	0.38^{b} ± 0.03	0.000	0.92^{b} ± 0.13	0.04^{b} ± 0.01	0.69^{b} ± 0.11	0.08^{c} ± 0.01	1.73
LE 11	30-Oct	0.27^{b} ± 0.01	0.36^{b} ± 0.01	0.000	0.99^{b} ± 0.08	0.04^{b} ± 0.00	0.67^{b} ± 0.07	0.08^{c} ± 0.01	1.78

Table S2. The precision and intra-/interday repeatability of the HPLC method for determination of chlorogenic acids and anthocyanins in extracts.

Analyte	Conc. (mg/mL)	Measured Conc. (mg/mL) [a]	RSD (%) [a]	Intraday RSD (%) [b]	Interday RSD (%) [c]
CGA	0.050	0.049	1.76	1.81	1.89
	0.100	0.098	1.11	1.24	1.34
	0.200	0.192	1.18	1.09	1.22
CyaGal	0.010	0.010	1.33	1.36	1.55
	0.100	0.098	1.11	1.32	1.61
	0.200	0.201	0.97	1.10	1.31

[a]Presission analysis (n=5), [b] the analyses were carried out at 0, 3, and 8 hrs on the same day, [c] the analyses were carried out next day.

Table S3. The trueness and stability assessment (each concentration was analyzed in triplicate (n=3))

Analyte	Original Conc.* (mg/mL)	Spiked Conc. (mg/mL)	Found Conc.* (mg/mL)	Recovery (%)	Found Conc. after 24h* (mg/mL)	Recovery after 24h (%)
			Green fruits LS2			
nCGA	0.031	0.020	0.0515	102.5± 0.2	0.051	99.0± 0.1
CGA	0.023	0.020	0.0425	97.5± 0.2	0.042	97.0± 0.1
CyaGal	0.000	0.020	0.0195	97.5± 0.2	0.019	95.0± 0.1
CyaAra	0.000	0.020	0.0205	102.5± 0.2	0.020	97.5± 0.1
CyaGlu	0.000	0.020	0.0195	97.5± 0.1	0.019	95.0± 0.1
			Red fruits LS7			
nCGA	0.027	0.020	0.0698	99.0± 0.1	0.070	97.5± 0.2
CGA	0.040	0.020	0.1205	102.5± 0.2	0.120	100.5± 0.1
CyaGal	0.067	0.020	0.2197	98.5± 0.1	0.219	95.0± 0.2
CyaAra	0.0138	0.020	0.0297	98.5± 0.2	0.029	95.5± 0.1
CyaGlu	0.0088	0.020	0.1198	99.0± 0.1	0.119	95.0± 0.1

[a]Average values
